# Genome‐wide association analysis of salinity responsive traits in *Medicago truncatula*

**DOI:** 10.1111/pce.13508

**Published:** 2019-02-27

**Authors:** Yun Kang, Ivone Torres‐Jerez, Zewei An, Veronica Greve, David Huhman, Nicholas Krom, Yuehua Cui, Michael Udvardi

**Affiliations:** ^1^ Noble Research Institute Ardmore Oklahoma 73401; ^2^ State Center for Rubber Breeding and Rubber Research Institute Danzhou Hainan 571700 China; ^3^ College of Biological Sciences University of Minnesota Huntsville Alabama 35806; ^4^ Department of Statistics and Probability Michigan State University East Lansing Michigan 48824

**Keywords:** GWAS, legume, *Medicago truncatula*, proline, salinity, SNP, vesicle trafficking

## Abstract

Salinity stress is an important cause of crop yield loss in many parts of the world. Here, we performed genome‐wide association studies of salinity‐stress responsive traits in 132 HapMap genotypes of the model legume *Medicago truncatula*. Plants grown in soil were subjected to a step‐wise increase in NaCl concentration, from 0 through 0.5% and 1.0% to 1.5%, and the following traits were measured: vigor, shoot biomass, shoot water content, leaf chlorophyll content, leaf size, and leaf and root concentrations of proline and major ions (Na^+^, Cl^−^, K^+^, Ca^2+^, etc.). Genome‐wide association studies were carried out using 2.5 million single nucleotide polymorphisms, and 12 genomic regions associated with at least four traits each were identified. Transcript‐level analysis of the top eight candidate genes in five extreme genotypes revealed association between salinity tolerance and transcript‐level changes for seven of the genes, encoding a vacuolar H^+^‐ATPase, two transcription factors, two proteins involved in vesicle trafficking, one peroxidase, and a protein of unknown function. Earlier functional studies on putative orthologues of two of the top eight genes (a vacuolar H^+^‐ATPase and a peroxidase) demonstrated their involvement in plant salinity tolerance.

## Abstract 2

Genome wide association studies (GWAS) of multiple physiological and biochemical traits related to salinity‐stress adaptation in *M. truncatula* were carried out using 2.5 million SNPs. Correlation analysis among the traits showed that salinity tolerance in the population is negatively correlated to sodium, chloride, and proline concentration in the leaf, while root proline levels were positively associated with salinity‐stress tolerance. GWAS analyses were performed with a mixed linear model, and 12 genomic regions that are associated with multiple traits each were identified; these regions harbor a total of 214 SNPs and 74 genes. These genomic regions were confirmed by a GWAS analysis using PC1 as a virtual trait, generated from principle component analysis of the 12 traits that are tightly correlated. Significant linkage disequilibrium was observed among these SNPs, possibly indicating co‐evolution of salinity tolerance alleles. Examination of gene expression of eight top suspect genes revealed associations between salinity tolerance and transcript level changes under salinity stress. Earlier functional studies on orthologues of two of the top eight genes (a vacuolar H^+^−ATPase and a peroxidase) confirmed their involvement in plant salinity tolerance in other species. Functional annotation of potential salinity tolerance genes points to the importance of transcriptional regulation, vesicle trafficking, and ROS scavenging under saline conditions.

## 1 INTRODUCTION

Salinity is an important abiotic stress that restricts crop distribution and reduces agricultural yield. It is estimated that over 6% of the world's total land area is affected by excess salts (Smajgl et al., 2015), and approximately 20% of arable land in more than 100 countries is affected by salinity (Sairam & Tyagi, 2004). Increasing salinity tolerance in crops will help to ensure food, feed, and industrial feedstock production on salt‐affected land.

The fundamental mechanisms of how plants sense and respond to salinity stress in both glycophytes and halophytes have been studied extensively, but remain incompletely understood. Multiple transporters and channels such as the Na^+^/H^+^ antiporter SOS1, the Na^+^/H^+^ exchanger NHX, the high affinity potassium transporter HKT1, as well as nonselective cation channels, have been shown to play important roles in maintaining cellular and plant‐level ion homeostasis under salinity stress (Julkowska & Testerink, 2015; Keisham, Mukherjee, & Bhatla, 2018). In addition, important genes involved in the signal transduction pathways that respond to salinity have been identified, including calcium‐dependent protein kinases (CDPKs), calcineurin B‐like proteins (CBLs), CBL‐interacting protein kinases (CIPKs), and mitogen‐activated protein kinases (MAPKs) (Shabala, Wu, & Bose, 2015).

To minimize the ionic stress caused by Na^+^ and Cl^−^, cells exclude and/or remove these ions from their cytoplasm via transporters, which can result in osmotic stress. To alleviate such stress, plant cells synthesize compatible solutes such as proline, glycine betaine, and soluble sugars that help them to retain water when ion levels in the apoplast or intracellular compartments are high. Proline also acts as a reactive oxygen species (ROS) scavenger and molecular chaperone to stabilize proteins and bio‐membranes under stress (Ashraf & Foolad, 2007; Matysik, Alia, & Mohanty, 2002). Proline biosynthesis serves as a redox buffer by consuming two NADPH per proline molecule, which helps to utilize excess electrons generated in the chloroplast under stress (Hare & Cress, 1997). Although generally regarded as a beneficial osmoticum, the link between proline and stress tolerance is somewhat unclear. In some studies, proline was found to accumulate more in tolerant plant genotypes than in sensitive genotypes under salt stress, consistent with an active role in stress tolerance (Jain, Nainawatee, Jain, & Chowdhury, 1991; Misra & Gupta, 2005; Ranganayakulu, Veeranagamallaiah, & Sudhakar, 2013). In other studies, however, proline levels are not positively correlated with salinity tolerance, but instead appear to increase as a consequence of cellular damage (Aziz, Martin‐Tanguy, & Larher, 1998; Lacerda, Cambraia, Oliva, & Ruiz, 2003; Moftah & Michel, 1987). Nonetheless, at least one genome‐wide association study (GWAS) has been performed to identify genes controlling proline level under dehydration stress in *Arabidopsis* (Verslues, Lasky, Juenger, Liu, & Kumar, 2014).

In leaves, high Na^+^ concentrations cause stomatal closure due to the osmotic effect of the solute, which consequently causes a reduction in the rates of photosynthesis and growth (Brugnoli & Lauteri, 1991; Munns & Tester, 2008). Another consequence of decreased stomatal aperture is that intercellular CO_2_ limitation causes NADP^+^ pool depletion, photorespiration enhancement, and ROS accumulation (Hossain & Dietz, 2016). Although important as signaling molecules, excess ROS may trigger chlorophyll degradation and, ultimately, cell death (Miller, Suzuki, Ciftci‐yilmaz, & Mittler, 2010; Suzuki, Koussevitzky, Mittler, & Miller, 2012). To scavenge ROS, antioxidant enzymes are positively regulated under salinity stress, for example, peroxidases, superoxide dismutase (SOD), catalase (CAT), etc. (Hossain & Dietz, 2016). Given the redox challenges of photosynthetic cells under stress, leaf and shoot growth are generally more sensitive to salinity stress than root growth (Hamaji et al., 2009; Munns, Passioura, Guo, Chazen, & Cramer, 2000).

To improve plant salinity tolerance, multiple approaches have been pursued. Buoyed by discovery of genes involved in plant salinity responses, genetic engineering has been used to generate a variety of transgenic plants in attempts to increase stress tolerance. Although positive outcomes have repeatedly been reported for transgenic plants under controlled‐growth conditions, less success has been found under field conditions (Fita, Rodríguez‐Burruezo, Boscaiu, Prohens, & Vicente, 2015; Flowers, 2004; Hanin, Ebel, Ngom, Laplaze, & Masmoudi, 2016). On the other hand, conventional breeding for salinity tolerance, through selection and introgression, has met with limited success in rice and wheat, possibly because of the complex nature of this trait (Ashraf & Foolad, 2013; Munns, James, & Läuchli, 2006; Varshney, Bansal, Aggarwal, Datta, & Craufurd, 2011).

In recent years, with advances in next generation sequencing technology, great progress has been made in identifying quantitative trait loci (QTL), associated with salt tolerance (Ashraf & Foolad, 2013; Hamwieh et al., 2011; Thomson et al., 2010). For example, a Na^+^/H^+^ antiporter (GmCHX1) was identified as a major salt‐tolerance gene in the soybean wild accessions by combining whole‐genome sequencing with QTL mapping (Qi et al., 2014). Later, GWAS indicated that this gene is a major contributor to the variance in salinity sensitivity among multiple soybean ecotypes; a conclusion supported by marker‐assisted selection that resulted in salt‐tolerant lines of soybean (Do et al., 2016; Patil et al., 2016; Zeng et al., 2017). Because cultivated soybean genotypes that contain nonfunctional GmCHX1 are generally very sensitive to salinity stress, it was postulated that loss of function of this gene in soybean may improve growth and seed production in nonsaline environments (Qi et al., 2014). Similar studies in *Arabidopsis* identified the sodium transporter, AtHTK1, as a major contributor to variance in shoot sodium accumulation under salinity stress in wild populations (Baxter et al., 2010; Busoms et al., 2015; Rus et al., 2006). Another GWAS study in *Arabidopsis* (Julkowska et al., 2016) indicated that a kinase, LRR‐KISS (Leucine‐Rich Repeat Kinase family protein Induced by Salt Stress), underlies variance in plant growth under salinity stress.

Apart from the progress noted above for soybean and *Arabidopsis*, little is known about the genes shaping natural variation in salinity tolerance in other species. A few traits affected by salinity stress have been investigated via GWAS with high density markers, including traits related to seed germination, growth rate, transpiration rate, and tissue Na^+^/K^+^ contents in rice (Al‐Tamimi et al., 2016; Patishtan, Hartley, Fonseca de Carvalho, & Maathuis, 2017; Shi et al., 2017), and root growth in *Arabidopsis* (Kobayashi et al., 2016). However, no overlapping genes or common mechanisms have been identified among these studies, and none of the identified genes have been validated with respect to salinity tolerance. Apart from soybean, no GWAS analysis of salinity traits with high‐density markers has been performed in other legumes.

*Medicago truncatula* is a model legume species for genetic and genomic research (Barker et al., 1990; Kang, Li, Sinharoy, & Verdier, 2016; Young & Udvardi, 2009). Since the launch of the *M. truncatula* HapMap (Haplotype Map) project in 2000, 262 genotypes have been sequenced and the resulting single nucleotide polymorphisms (SNP) information released (Young et al., 2011). In the current study, we characterized a spectrum of salinity stress‐related traits in a diverse subset of the *M. truncatula* HapMap panel consisting of 132 *M. truncatula* genotypes. Plants were grown in soil and were subjected to a step‐wise increase in NaCl concentration, from 0 through 0.5% and 1.0% to 1.5%. Traits that were measured in salt‐stressed (and control) plants included a qualitative, visual salt‐tolerance score, relative shoot biomass reduction (%), leaf chlorophyll content reduction (%), leaf size reduction (%), shoot water content (%), and leaf and root concentration of proline and major ions (Na^+^, Cl^−^, K^+^, Ca^2+^, Mg^2+^, NH_4_^+^, PO_4_^3+^, SO_4_^2−^, NO_3_^−^, and malate). GWAS was carried out using 2.5 million high‐quality SNP markers. By performing GWAS on complex, emergent traits like growth/biomass as well as on potentially‐underlying, fundamental traits or “phenes” such as ion and metabolite concentrations, which reflect cellular ion homeostasis and metabolism, we hoped to break‐down salinity tolerance into its component parts while at the same time identifying genetic loci for tolerance in a more refined and robust manner.

## 2 MATERIALS AND METHODS

### 2.1 Plant material

Seeds of all the lines used in this study were obtained from the *M. truncatula* HapMap germplasm resource center (http://www.medicagohapmap.org/hapmap/germplasm). One hundred thirty‐two lines (Table S1) were selected based on population structure for maximum variance from the 220 lines that were used previously for the GWAS of drought‐related traits (Kang et al., 2015).

*M. truncatula* seeds were scarified on sand paper (p800) and germinated on wet filter paper for 48 hr at 4°C by overnight storage in the dark at room temperature, followed by planting. Plants were grown individually in 2″ × 7″ plastic cones (Stuewe & Sons Inc.) containing a 4:1 (v/v) mixture of Metromix 350 and sand, in a growth chamber with 16 hr day/8 hr night, 22°C, and 40% relative humidity. For each experiment, 12 seedlings were planted for each line, six for control conditions and six for salinity‐stress treatment. The plants were placed in the growth chamber in a randomized complete block design. Light density (a combination of fluorescent and incandescent) at plant level was approximately 200 μmol m^−2^ s^−1^. Plants were watered with one fourth B&D nutrient solution (Broughton & Dilworth, 1971) containing 8 mM of N (2 mM KNO_3_, 3 mM NH_4_NO_3_). Three replicates were performed for the entire experiment.

### 2.2 Salinity stress treatment

Ten days after seedling transfer to pots (day 10), plants were watered with 0.5% NaCl (86 mM) in one half B&D nutrient solution, which was applied to both the tray holding the cones and the top of the soil with a squeeze bottle to avoid leaf damage. Excess solution in the trays was removed 2 hr after the salinity solution treatment. Sodium chloride concentration in the nutrient solution was increased to 1.0% (172 mM) at day 15 then 1.5% (257 mM) at day 20, in the same way. No extra watering between treatments was necessary because of reduced transpiration. Roots and shoots were harvested 5 days following the 1.5% NaCl treatment.

### 2.3 *In‐vivo* leaf chlorophyll content measurement

Four days following the 1.5% NaCl treatment, *in‐vivo* leaf chlorophyll content was measured on salt‐stressed and control plants, using a Chlorophyll Meter SPAD‐502plus (Spectrum Technologies Inc., http://www.specmeters.com/). The terminal leaflet of the uppermost fully‐expanded leaf was used for the measurement. Each leaflet was measured twice at different positions, avoiding the midrib and edges, and the average of the two readings was recorded.

### 2.4 Leaf size measurement and plant harvest

Five days following the 1.5% NaCl treatment, the two youngest fully‐expanded leaves, from both control and NaCl‐treated plants, were harvested and immediately used for individual leaf size measurement with the Li‐3000A (LI‐COR) portable area meter. These two leaves were then flash‐frozen in liquid nitrogen, and stored at −80°C until being dried at −40°C in a lyophilizer. After the two youngest fully‐expanded leaves were harvested, the rest of the shoot was harvested separately. Shoots were dried in a 55°C oven and weighed. Total shoot dry weight was the sum of the two youngest fully‐expanded leaves and the rest of the shoot. Roots were harvested at the same time as the shoots, rinsed well, flash‐frozen in liquid nitrogen, and then stored at −80°C until being dried at −40°C in a lyophilizer. Dried tissues were ground to powder in a bead beater (BioSpec, https://www.biospec.com/).

### 2.5 Tissue ion (including malate) measurement

Approximately 5 mg of dry ground leaf or root tissue (six plants pooled together) was dissolved in 5–14 ml of MQ water, vortexed, and then shaken at 200 rpm for 1 hr. The solution was then filtered through a 0.2 μm nylon filter (F13–2020, VWR) and the filtrate was used for measurement of total ions with ion chromatography. Chromatographic separation was achieved on a Thermo Scientific ICS‐5000 IC system (Thermo Fisher Scientific, USA) using a Dionex CS12A Ion Pac analytical column with a AG12A guard column for cations, or a Dionex AS11HC analytical column with a AG11HC guard column for anions. The cation eluent source was a Thermo Scientific Dionex EGC III Methane sulphonic acid eluent generator cartridge. The anion eluent source was Thermo Scientific Dionex EGC KOH cartridge. Standard curves were prepared using dilutions of Thermo Scientific Dionex Seven Anion Standard II and Six Cation Standard II. Malate standard was prepared separately and then mixed with the Anion Standard. Quantification was achieved using software Chromeleon 7.2 version SR4.

### 2.6 Tissue proline quantification

Tissue proline levels were analyzed with a biochemical assay modified from Bates et al (Bates, Waldren, & Teare, 1973) and Hamid et al (Hamid et al., 2003). Proline concentration was determined using a standard curve using L‐proline.

### 2.7 Filtering of SNPs

SNPs were filtered with TASSEL 5.2.7 (Bradbury et al., 2007), with a minimum allele frequency of 5% and minimum counts of 112 (85% of 132). Missing SNPs were not imputed because of the high density of the existing SNPs. After filtering, a total of 2,528,531 SNPs remained and were used in the association analysis.

### 2.8 Cladogram

The cladogram tree was generated in TASSEL 5.2.7 (Bradbury et al., 2007) with the neighbor‐joining method using 40,000 randomly selected SNPs (5,000 SNPs each chromosome).

### 2.9 Q matrix deduction

Q matrix was deduced with the STRUCTURE program (Pritchard, Stephens, & Donnelly, 2000) using 40,000 SNPs (5,000 random SNPs from each chromosome). We evaluated K = 1–9 to infer the optimal value of K (i.e., the number of clusters) from the simulation summary using the methods of Pritchard et al. (2000) and Evanno, Regnaut, and Goudet (2005).

### 2.10 Genome‐wide genotype–phenotype association analysis

The least square means of the phenotypic data collected in three replicates were calculated in R with library “lsmeans” and used in GWAS. The proline and ion measurements were carried out only on the last replicate, and the standard‐calibrated values obtained were used directly in GWAS. The mixed linear model (MLM) and general linear model (GLM) embedded in TASSEL (Bradbury et al., 2007) were used to test for association between SNPs and phenotypic traits. For the MLM analyses, a Kinship matrix was calculated with centered‐IBS in TASSEL (Bradbury et al., 2007), and no compression was applied. Quantile–quantile (QQ) plots were generated in R (for GLM and MLM‐Q) or using 10% of all SNPs randomly‐selected from the eight chromosomes in TASSEL (for MLM + Q). Linkage disequilibrium among SNPs was also performed in TASSEL 5.2.7 (Bradbury et al., 2007).

### 2.11 Total RNA extraction and qRT‐PCR

Total RNA from shoot and root was extracted using RNeasy Plant Mini Kit (Qiagen) followed by genomic DNA removal and column purification. Reverse transcription was performed with oligo dT_20_ and Super Script III Reverse Transcriptase as described previously (Kakar et al., 2008). Amplification of templates followed standard PCR protocols with SYBR Green‐based detection system. Transcript levels were normalized using the geometric average of three housekeeping genes, *Ubiquitin‐Conjugating enzyme E2* (TC106312), *Polypyrimidine Tract‐Binding protein* (TC111751), and *Ubiquitin* (TC102473). The primers used for all the target and housekeeping genes are listed in Table S6.

### 2.12 Statistical analysis

Principal component analysis (PCA) was performed in R using the “PCA” function in the package “FactoMineR” (Lê, Josse, & Husson, 2008). Association analysis among traits was performed in SAS 9.4 (SAS Institute, Cary, NC) using the CORR procedure. False discovery rate (*q* value) was calculated using the R package “qvalue” (Storey & Tibshirani, 2003). Significant tests were performed in Excel 2013 using student's *t*‐test, two‐tails, assuming equal variance.

## 3 RESULTS

### 3.1 Phenotypic data collection and correlations among traits

**Figure 1:**
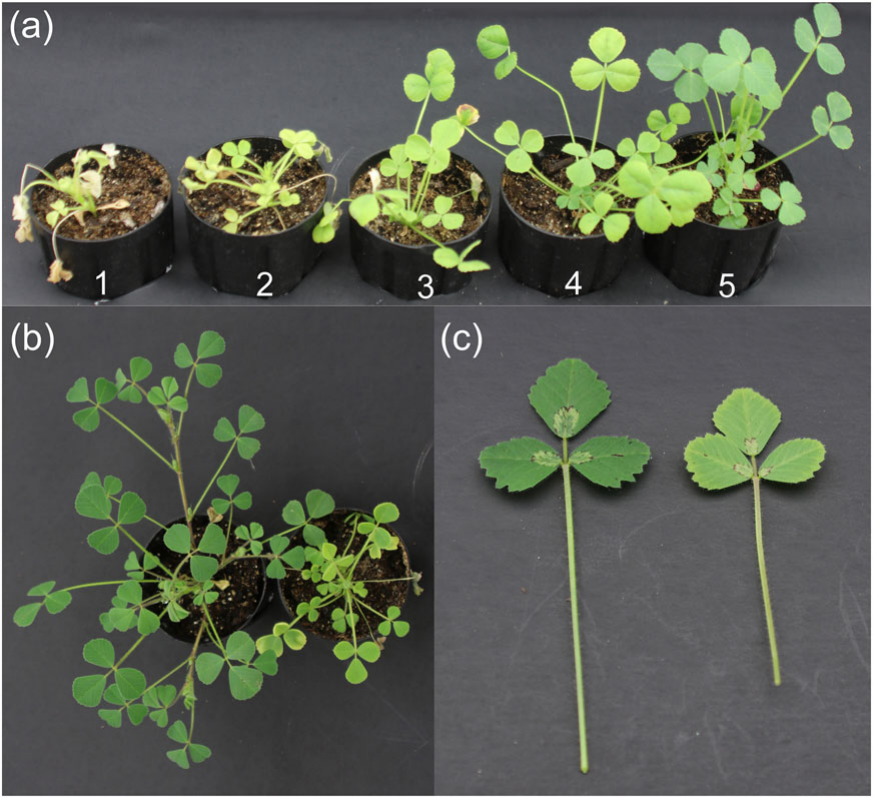
Effect of salinity on *M. truncatula* growth. (a) stressed *M. truncatula* plants with tolerance scores from 1 to 5. (b) Entire plant. (c) Leaves. In b and c, the plant/leaf on the left was non‐stressed. All photos were taken 4 days after the 1.5% NaCl treatment, 1 day before plant harvest

**Figure 2:**
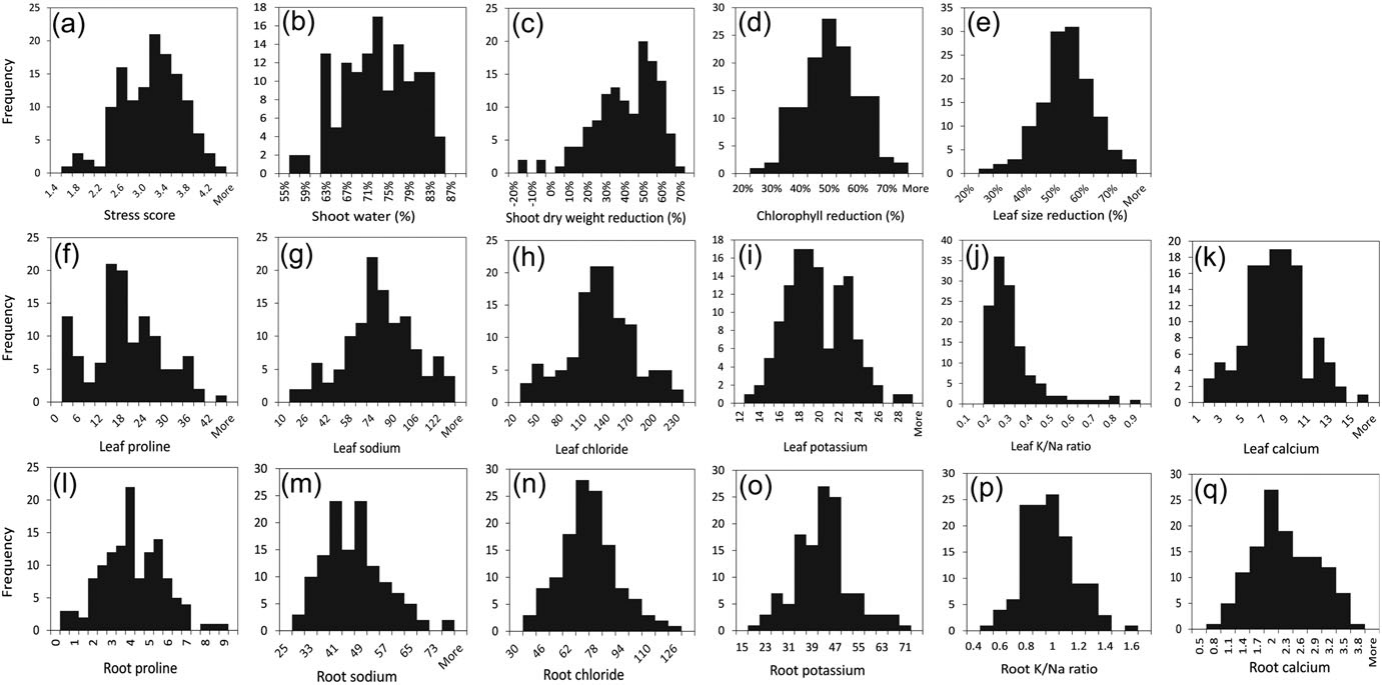
Distribution of salinity stress‐related traits in a collection of 132 *M. truncatula* lines/ecotypes. Traits are indicated on the x‐axis and number of lines on the y‐axis

Preliminary experiments with 30 *M. truncatula* HapMap lines were carried out to establish a salinity stress regime that affected plant growth without overwhelming plants completely. As a result, a protocol with sequential increases in NaCl concentration in the nutrient solution was chosen: 0.5% NaCl (86 mM) for 5 days, 1% (171 mM) for 5 days, and 1.5% (257 mM) for 5 days. These treatment conditions resulted in significant leaf chlorosis, leaf size reduction, and growth retardation compared with non‐treated control plants (Figure 1). On the basis primarily of plant vigor and the degree of leaf chlorophyll loss, we scored stressed plants from 1 to 5, with 5 being the most tolerant (Figure 1a). In addition, shoot biomass, leaf chlorophyll content, and leaf size were measured for both treated and controlled plants, whereas shoot water content, leaf and root concentrations of proline and major ions (Na^+^, Cl^−^, K^+^, Ca^2+^, Mg^2+^, NH_4_^+^, PO_4_^3+^, SO_4_^2−^, NO_3_^−^, malate) were measured for treated plants only. For most of these traits, a near normal distribution was observed; leaf K^+^/Na^+^ ratio was an exception (Figure 2). Notably, concentrations of sodium, chloride, calcium, and proline were much higher in leaves than roots of NaCl‐treated plants, whereas potassium concentration exhibited the opposite response to salinity (Figure 2g–i,m–o; Figure S1). As a consequence, the K^+^/Na^+^ ratio was approximately 2‐fold higher in the roots than in the leaves (Figure 2j,p).

**Figure 3:**
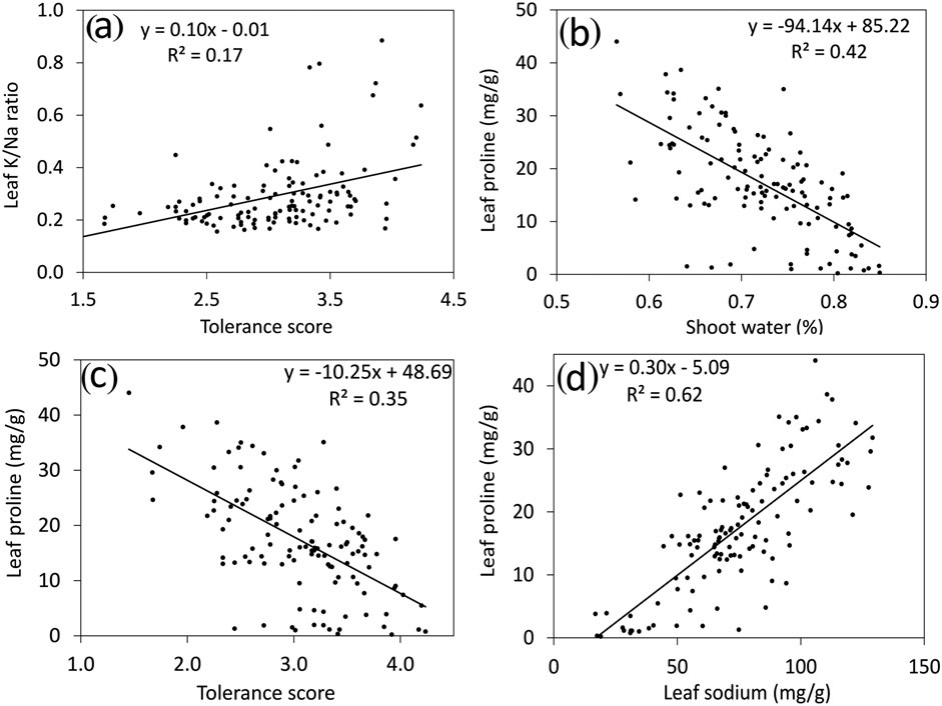
Correlation between salinity‐related traits. Average measurements of 132 *M. truncatula* lines are plotted for each trait. Significant correlations (*P* < 0.001) were found for all comparisons shown

To reveal relationships between traits, correlation analyses were performed. In general, shoot and leaf traits were positively correlated but, with the exception of proline concentration, shoot and root traits were less correlated (Table 1). Relatively strong and significant correlations were found between all of the following traits: shoot tolerance scores; reduction in leaf size, shoot dry weight, and leaf chlorophyll; leaf concentrations of sodium, chloride, and proline; and water content of the shoot (Table 1). Compared with chloride, sodium, calcium, and the K/Na ratio, potassium concentration in leaves were less tightly correlated to tolerance scores (*r* = −0.35). Sodium and chloride concentrations were extremely highly correlated to each other, both in leaves and roots, with correlation coefficients of 0.99 and 0.89, respectively. Interestingly, proline levels in leaves were negatively correlated to tolerance scores and shoot water contents, but were positively correlated to leaf sodium concentrations, whereas root proline levels were positively correlated with salinity tolerance (Figure 3). Thus, salinity‐tolerant *M. truncatula* genotypes tended to accumulate less proline in the leaf but more proline in the root under salinity stress, compared with the sensitive genotypes.

**Table 1:** Correlation coefficients (r) of salinity‐stress related traits

	ST_DWred	LF_ChlRed	LF_SizeRed	ST_Water%	LF_Proline	LF_Sodium	LF_Chloride	LF_Potassium	LF_KNaRatio	LF_Calcium	RT_Proline	RT_Sodium	RT_Chloride	RT_Potassium	RT_KNaRatio	RT_Calcium
**ToleranceScore**	**−0.60** ***	**−0.70** ***	**−0.60** ***	**0.74** ***	**−0.59** ***	**−0.55** ***	**−0.55** ***	**−0.35** ***	**0.41** ***	**−0.41** ***	**0.33** ***	0.027	0.10	0.030	0.026	0.065
**ST_DWred**		**0.41** ***	**0.59** ***	**−0.60** ***	**0.45** ***	**0.40** ***	**0.42** ***	0.15735	**−0.32** ***	**0.38** ***	**−0.18** *	−0.14	−0.15	−0.058	0.082	−0.0066
**LF_ChlRed**			**0.46** ***	**−0.52** ***	**0.41** ***	**0.46** ***	**0.45** ***	**0.26** **	**−0.29** **	**0.32** ***	**−0.33** ***	−0.021	−0.12	−0.051	0.0042	−0.12
**LF_SizeRed**				**−0.67** ***	**0.53** ***	**0.53** ***	**0.54** ***	**0.32** ***	**−0.35** ***	**0.46** ***	**−0.33** ***	−0.098	−0.16	−0.17	−0.15	−0.0031
**ST_Water%**					**−0.65** ***	**−0.60** ***	**−0.61** ***	**−0.34** ***	**0.48** ***	**−0.51** ***	**0.32** ***	0.14	**0.20** *	0.15	0.087	0.029
**LF_Proline**						**0.78** ***	**0.79** ***	**0.41** ***	**−0.63** ***	**0.56** ***	**−0.38** ***	0.022	−0.057	−0.0045	−0.070	0.033
**LF_Sodium**							**0.99** ***	**0.46** ***	**−0.80** ***	**0.66** ***	**−0.47** ***	0.022	−0.070	−0.044	−0.13	−0.0069
**LF_Chloride**								**0.52** ***	**−0.78** ***	**0.73** ***	**−0.46** ***	0.029	−0.076	−0.085	**−0.18** *	0.0068
**LF_Potassium**									−0.069	**0.51** ***	**−0.25** **	0.046	−0.098	−0.092	−0.15	−0.034
**LF_KNaRatio**										**−0.53** ***	**0.23** *	0.013	0.022	0.051	0.11	−0.052
**LF_Calcium**											**−0.21** *	0.067	−0.052	**−0.22** *	**−0.35** ***	0.028
**RT_Proline**												−0.024	0.069	0.034	0.081	−0.027
**RT_Sodium**													**0.89** ***	**0.58** ***	**−0.34** ***	0.13
**RT_Chloride**														**0.72** ***	−0.11	**0.28** **
**RT_Potassium**															**0.51** ***	**0.29** ***
**RT_KNaRatio**																0.17

*Note*. Chl: chlorophyll; ST: shoot; LF: leaf; RT: root; Red: reduction (%).

* ,

** , and

*** indicate significant differences at *P* < 0.05, 0.01, and 0.001, respectively.

Several other ions were analyzed, and leaf magnesium, leaf sulfate, leaf ammonium, leaf nitrate, root malate, and root phosphate were found to be significantly correlated with salinity tolerance, either positively or negatively (Table S2).

### 3.2 Genome‐wide association analysis and GO enrichment of “top‐suspect” genes

**Figure 4:**
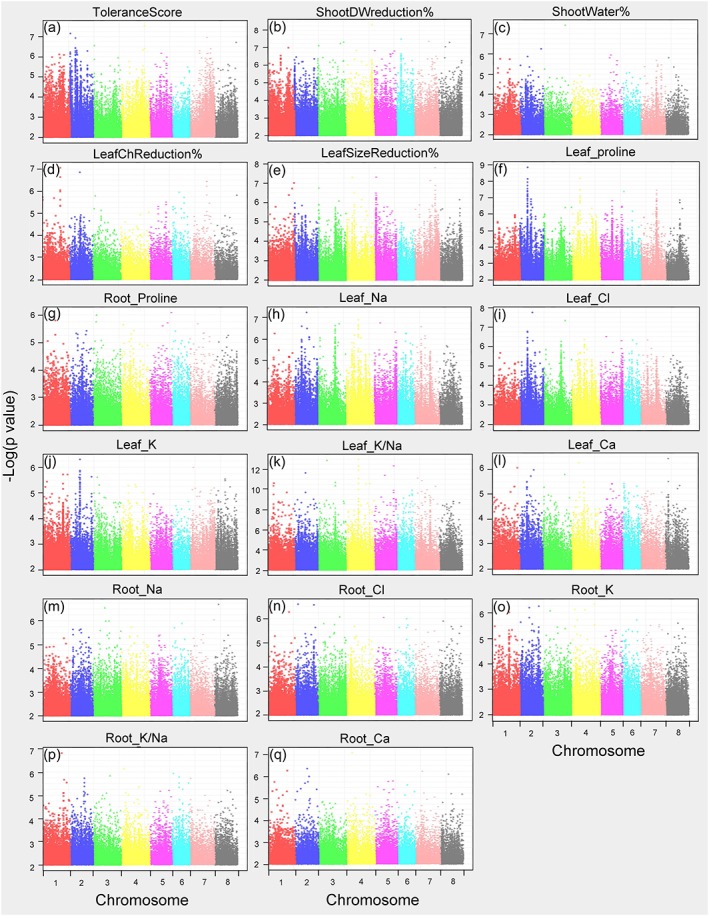
Manhattan plots (mixed linear model) of mapped single nucleotide polymorphisms (SNP) markers associating with each trait. Only SNPs with *P* values smaller than 0.01 are plotted

Genome‐wide association analysis using mixed linear model or general linear model was performed in TASSEL (Bradbury et al., 2007; Zhang et al., 2010). In the cladogram tree, the 132 lines used in the current study were clearly separated into two major groups (Figure S2). This is consistent with the cluster number determined by the program structure (Pritchard et al., 2000), which was also two (Figure S3). Because of the existence of population structure in the GWAS population, QQ plots generated with GLM or MLM without Q‐value correction were inflated for majority of the traits (Figure S4). To control for false positives, the population structure (Q value) was included in the MLM; GWAS Manhattan plots and QQ plots were generated and the lowest *P* values ranged from 10^−6^ to 10^−13^ (Figures 4, Figure S4). From the Manhattan plots, chromosome 2 appeared to be a “hot spot” for SNPs with lowest *P* values in multiple traits. For each trait, we collected the 200 SNPs with the lowest *P* values, the genes in each SNP's vicinity, as well as the closest *Arabidopsis* orthologues and *M. truncatula* microarray probe‐sets (Table S3).

To gain an overview of genes that may contribute to salinity tolerance, we performed gene ontology (GO) enrichment analysis of all genes in the vicinity of the 200 SNPs with the lowest *P* values for 14 traits (Table S3). We used the GO of *Arabidopsis* orthologues for this analysis because they are better curated than for *Medicago*. Among all the biological processes, “post‐embryonic development” was most‐enriched among the selected genes, with a false discovery rate (FDR) of 1.10E‐12. Genes in the categories “response to stress” and “response to stimulus” were also highly enriched (Figure S5).

### 3.3 Identification of potential causative SNPs and genes

**Figure 5:**
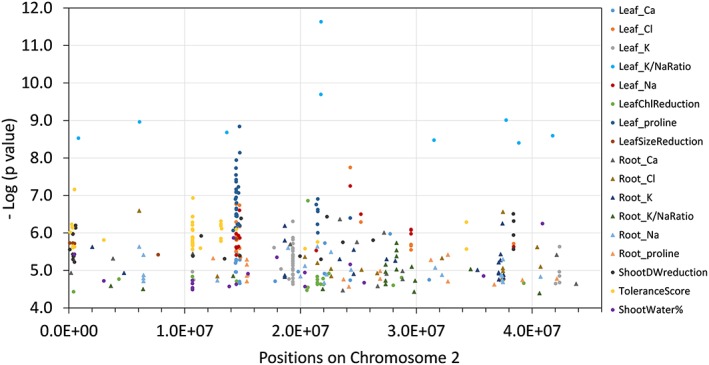
Top potential causative single nucleotide polymorphisms (SNPs) identified by genome‐wide association studies on chromosome 2. All SNPs shown are among the 100 SNPs with the lowest *P* values for each trait

**Figure 6:**
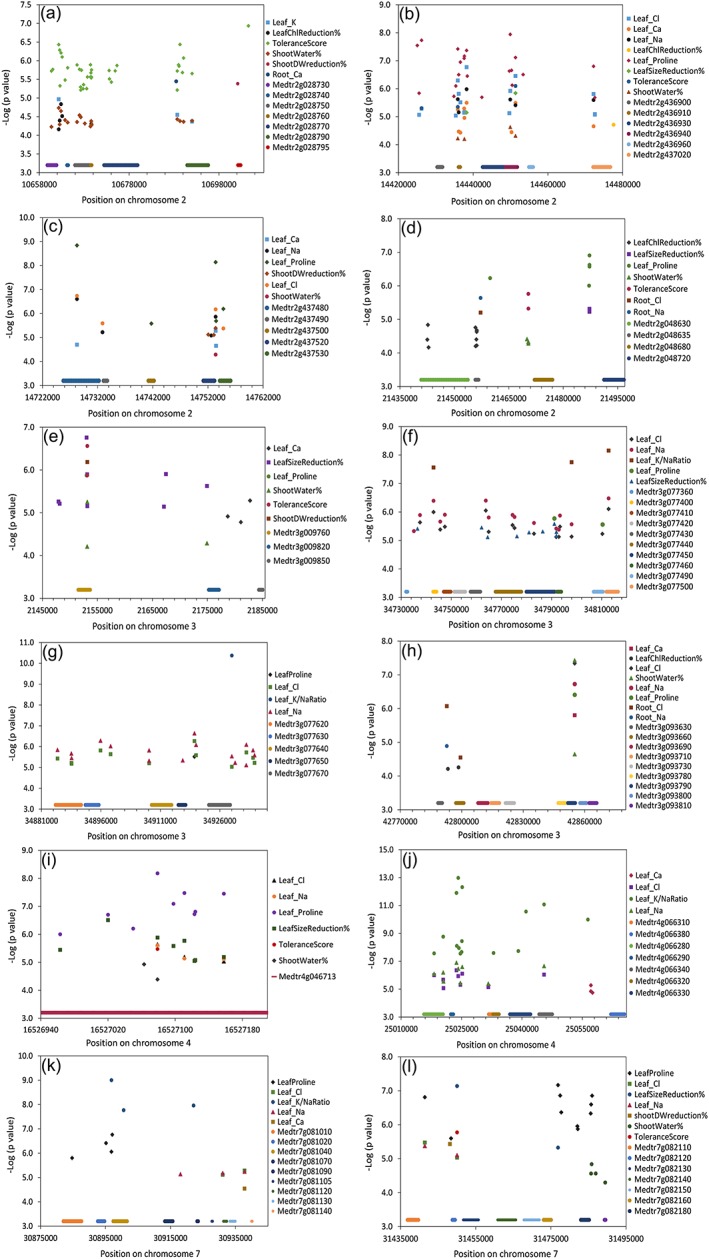
Top 12 genomic regions and predicted genes identified by genome‐wide association studies on chromosome 2 (a–d), chromosome 3 (e–h), chromosome 4 (i,j), and chromosome 7 (k,l). These genomic regions contain multiple low *P* value single nucleotide polymorphisms (SNPs) associated with at least four traits each. All SNPs shown are among the 200 SNPs with the lowest *P* values for each trait

**Figure 7:**
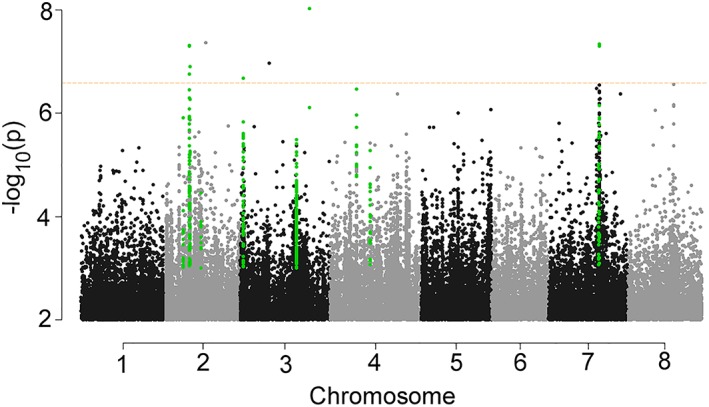
Manhattan plot (mixed linear model) of mapped single nucleotide polymorphism (SNP) markers associating with PC1 (55% explained variance) generated in the principal component analysis of the first 12 traits that are in tight correlation in Table 1. Only SNPs with *P* values smaller than 0.01 are plotted. SNPs reside within the 12 genomic regions (Figure 6) are highlighted. Dotted line indicates q value (FDR) cutoff 0.05

**Figure 8:**
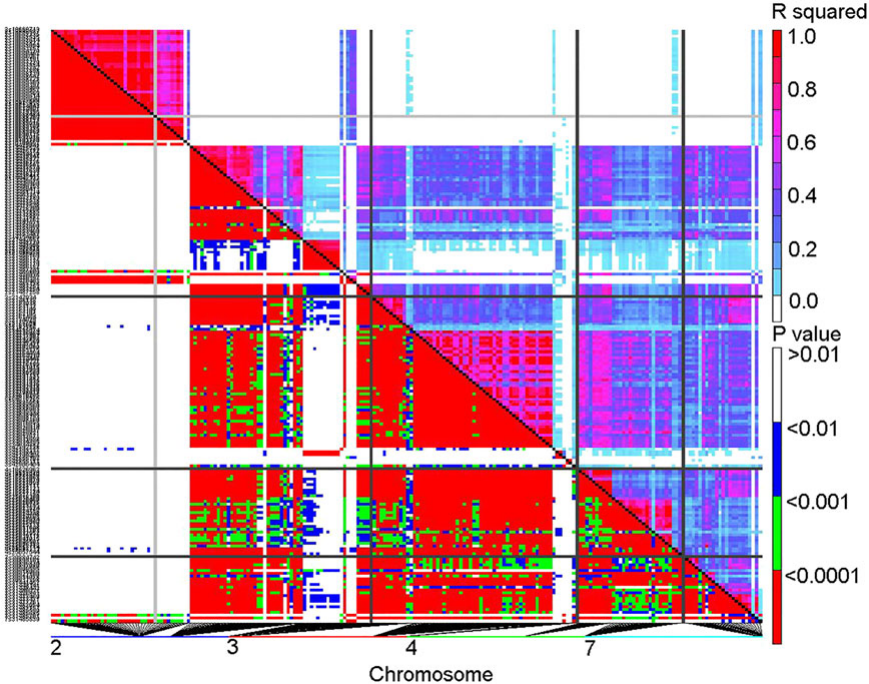
Linkage disequilibrium map of the 214 single nucleotide polymorphisms (SNPs) within the top 12 genomic regions shown in Figure 6

In analyzing the top 100 SNPs associated with each of the 14 traits, we found a large portion of them to be linked to multiple traits (Table S3), forming distinct “hotspots” on chromosomes especially on chromosome 2 (Figure 5). Considering the tight association among different traits (Table 1), we selected the top SNPs that were tightly associated with multiple traits, as well as the genomic regions that contained these SNPs. In doing so, we identified 12 genomic regions (QTLs) harboring SNPs that are in tight association with at least four traits (rank 200 or higher; Figure 6). In parallel, we performed PCA analysis of the first 12 traits (ToleranceScore, ST_DWred, LF_ChlRed, LF_SizeRed, ST_Water%, LF_Proline, LF_Sodium, LF_Chloride, LF_Potassium, LF_KNaRatio, LF_Calcium, and RT_Proline) that are tightly correlated in Table 1 and performed GWAS analysis using PC1 (that explained 55% of the variance) as a new trait, which identified the same 12 genomic regions as top hits (Figure 7). There are a total of 214 SNPs and 74 genes in these 12 regions (Figure 6). A total of 94 of the SNPs reside within genes. Linkage disequilibrium (LD) analysis revealed that the 214 SNPs in these 12 genomic regions are under tight LD within each region; the majority of these also show strong LD across different chromosomes (Figure 8).

Among all the regions, the most significant one, considering the ranking and quantity of top SNPs, is on chromosome 2 near position 14,450,000 (Figure 6b). This region is about 60 kb long and harbors 61 low *P* value SNPs identified in the association studies of eight different traits. In addition, nine of the 61 SNPs rank in the top 10 in the GWAS results of three traits (leaf calcium, leaf chloride, and leaf proline; Table S3). The central SNP is 2:14451314, which has low association *P*‐values in seven traits. A total of six genes are located in this region (Figure 6b; Table S3). In addition to this region on chromosome 2, three other regions were identified (Figure 6a,c,d). Furthermore, four regions on chromosome 3, as well as two regions each on chromosomes 4 and 7, were identified (Figure 6e‐l). It is worth noting that two genomic regions, one on chromosome 3 (3:2147934 to 3:2182756) and one on chromosome 4 (4:16526963 to 4:16527158), harbor a total of four genes, and all of them are involved in cellular redox homeostasis (three FAD‐linked oxidoreductases and one peroxidase family protein; Tables 2; Table S3). Other less significant regions are highlighted in Table S3.

**Table 2:** List of top suspect genes in the 12 selected genomic regions of Figure 6 that contain SNPs ranking 200 or less in the GWAS analyses of at least four traits. Genes in bold were characterized by qRT‐PCR for their response to the standard salinity treatment. Microarray data on gene expression under hydroponic salinity stress (up to 48 hr), drought stress, and seed desiccation were obtained from https://mtgea.noble.org/v3/index.php

Gene	Start	End	Strand	Annotation (Mt. 4.0)	*M. truncatula* Microarray probeset	In *M. truncatula*, regulation under			
						salinity stress?^a^	drought in root?^b^	drought in shoot?^b^	seed desiccation?^c^
Medtr2g028730	10659792	10661824	−	Trm112p‐like protein	Mtr.16016.1.S1_at	no	no	no	**
Medtr2g028740	10664184	10664396	−	hypothetical protein	none				
**Medtr2g028750**	**10665954**	**10669267**	**+**	**nonclathrin coat protein zeta1‐COP**	Mtr.16017.1.S1_at	no	no	**	no
Medtr2g028760	10669468	10669638	−	hypothetical protein	none				
Medtr2g028770	10672439	10679871	−	magnesium transporter CorA family protein	Mtr.52281.1.S1_at	**	***	***	**
**Medtr2g028790**	**10690943**	**10695600**	**+**	**synaptobrevin‐like protein**	Mtr.16019.1.S1_at	no	**	***	***
Medtr2g028795	10702242	10702842	−	hypothetical protein	none				
Medtr2g048630	21440873	21453740	+	regulator of nonsense transcripts‐like protein	Mtr.21538.1.S1_at	*	***	**	*
Medtr2g048635	21455804	21456661	+	pentatricopeptide (PPR) repeat protein	none				
Medtr2g048680	21472115	21477040	+	ankyrin repeat protein EMB506	Mtr.42601.1.S1_at	no	**	no	**
Medtr2g048720	21491260	21497003	−	inositol transporter 4	Mtr.48631.1.S1_at	***	***	**	***
**Medtr2g436900**	**14430380**	**14431772**	**−**	**C2 domain protein**	Mtr.12381.1.S1_at	**	*	no	*
Medtr2g436910	14436094	14436501	−	hypothetical protein	none				
Medtr2g436930	14442534	14448484	+	substrate carrier family protein	Mtr.9356.1.S1_at	no	*	***	**
**Medtr2g436940**	**14448712**	**14451781**	**−**	**hypothetical protein**	Mtr.41375.1.S1_at	no	***	**	***
Medtr2g436960	14454918	14456001	−	light‐harvesting complex I chlorophyll A/B‐binding protein	Mtr.15166.1.S1_at	*	*	no	*
**Medtr2g437020**	**14472093**	**14476696**	**−**	**mTERF protein**	none				
Medtr2g437480	14725811	14732214	+	chromatin remodeling complex subunit	none				
Medtr2g437490	14733099	14733710	−	transmembrane protein, putative	Mtr.21581.1.S1_at	no	no	no	no
Medtr2g437500	14741244	14742166	−	transcription factor GTE6, putative	none				
Medtr2g437520	14751210	14753146	+	hypothetical protein	none				
Medtr2g437530	14754185	14756065	−	DREPP plasma membrane protein	none				
**Medtr3g009760**	**2151533**	**2153673**	**+**	**FAD‐linked oxidoreductase**	Mtr.13518.1.S1_at	no	no	**	*
Medtr3g009820	2175256	2177107	+	FAD‐linked oxidoreductase	none				
Medtr3g009850	2184364	2185062	+	FAD‐linked oxidoreductase	none				
Medtr3g077360	34731943	34732457	+	hypothetical protein	none				
Medtr3g077400	34742781	34744107	−	hypothetical protein	none				
Medtr3g077410	34747003	34749760	+	NAD(P)‐binding rossmann‐fold protein	Mtr.51752.1.S1_at	no	no	no	*
Medtr3g077420	34751208	34755524	+	transcription factor, putative	Mtr.17177.1.S1_at	no	***	**	no
Medtr3g077430	34757686	34761543	−	carboxyl‐terminal peptidase	Mtr.51747.1.S1_at	***	***	**	*
Medtr3g077440	34767661	34777932	+	modifier OF SNC1 1, putative	Mtr.29563.1.S1_at	no	**	*	*
Medtr3g077450	34780007	34791257	+	modifier OF SNC1 1, putative	Mtr.14802.1.S1_at	no	**	no	*
Medtr3g077460	34792157	34793856	−	cytochrome P450 family 71 protein	Mtr.51055.1.S1_at	no	no	no	***
Medtr3g077490	34806841	34810528	+	embryo defective 2759 protein	Mtr.14799.1.S1_at	no	***	**	**
Medtr3g077500	34811954	34816546	+	transmembrane protein, putative	Mtr.39510.1.S1_s_at	no	*	no	no
Medtr3g077620	34884550	34891032	+	phox (PX) domain protein	Mtr.14792.1.S1_s_at	no	no	no	no
Medtr3g077630	34892075	34895483	−	transmembrane protein, putative	Mtr.4339.1.S1_at	*	no	***	**
Medtr3g077640	34908806	34913845	+	plastid phosphate translocator	none				
Medtr3g077650	34915543	34917386	+	transcription factor MYB98	Mtr.48147.1.S1_at	*	no	no	***
Medtr3g077670	34923242	34928630	−	boron transporter‐like protein	Mtr.48146.1.S1_s_at	no	**	*	**
Medtr3g093630	42788484	42790479	+	hypothetical protein	none				
Medtr3g093660	42797203	42800974	−	RING zinc finger protein, putative	none				
Medtr3g093690	42807993	42812754	+	transmembrane protein, putative	Mtr.6105.1.S1_at	no	**	*	no
Medtr3g093710	42814305	42818320	+	receptor‐like kinase	Mtr.45101.1.S1_at	**	*	**	**
Medtr3g093730	42821390	42825561	−	methylesterase	none				
Medtr3g093780	42847107	42851695	+	DUF3133 family protein	Mtr.33238.1.S1_at	no	no	no	no
Medtr3g093790	42851825	42855319	−	nucleic acid‐binding, OB‐fold‐like protein	Mtr.12947.1.S1_at	no	no	**	**
Medtr3g093800	42857714	42860884	−	transcription elongation factor S‐II, putative	Mtr.43413.1.S1_at	*	**	*	no
Medtr3g093810	42862192	42865834	−	ubiquinol‐cytochrome C reductase complex protein, putative	Mtr.37864.1.S1_at	no	***	no	***
Medtr3g093900	42905400	42907112	+	MADS‐box transcription factor family protein, putative	none				
**Medtr4g046713**	**16525346**	**16527516**	**−**	**peroxidase family protein**	Mtr.44569.1.S1_at	***	***	no	no
Medtr4g066280	25015686	25020396	−	glycoside hydrolase family 1 protein	Mtr.47870.1.S1_at	*	no	no	no
Medtr4g066290	25022356	25022951	+	Nodule Cysteine‐Rich (NCR) secreted peptide	none				
Medtr4g066310	25031757	25032810	−	hypothetical protein	none				
Medtr4g066320	25032880	25034289	+	hypothetical protein	none				
Medtr4g066330	25037019	25042115	−	glycoside hydrolase family 1 protein	none				
Medtr4g066340	25044288	25047517	−	cyanogenic beta‐glucosidase, putative	none				
Medtr4g066380	25062108	25065460	−	basic helix loop helix (bHLH) DNA‐binding family protein	none				
**Medtr7g081010**	**30882053**	**30887619**	**−**	**archaeal/vacuolar‐type H + ‐ATPase subunit B**	Mtr.12301.1.S1_at	***	***	**	***
Medtr7g081020	30892633	30894709	+	protein phosphatase 2C family protein	Mtr.9338.1.S1_at	**	***	***	***
Medtr7g081040	30897385	30901657	−	trafficking protein particle complex subunit‐like protein	Mtr.42623.1.S1_at	no	***	**	*
Medtr7g081070	30913276	30915702	−	DUF1279 family protein	none				
Medtr7g081090	30923192	30923518	+	transmembrane protein, putative	none				
Medtr7g081105	30927714	30927950	+	Thionin related	none				
Medtr7g081120	30931309	30932323	+	transmembrane protein, putative	none				
Medtr7g081140	30939953	30940153	−	hypothetical protein	none				
Medtr7g082110	31436869	31439723	+	receptor‐like kinase, putative	none				
Medtr7g082120	31448827	31449485	−	hypothetical protein	none				
Medtr7g082130	31451724	31455731	−	hypothetical protein	none				
Medtr7g082140	31460852	31465655	−	peptidase M50B‐like protein	Mtr.44385.1.S1_at	no	***	no	***
Medtr7g082150	31467830	31471924	−	U1 small nuclear ribonucleoprotein	Mtr.33442.1.S1_at	*	**	*	no
Medtr7g082160	31473081	31475033	−	PPR containing plant‐like protein	none				
Medtr7g082180	31482984	31485168	+	hypothetical protein	Mtr.39860.1.S1_at	**	no	**	*
Medtr7g082190	31489286	31489554	+	transmembrane protein, putative	none				

*Note*.

a Li et al. (Li et al., 2009);

b Zhang et al. (Zhang et al., 2014);

c Verdier et al. (Verdier et al., 2012).

* ,

** , and

*** indicate significant differences at p < 0.05, 0.01 and 0.001, respectively.

### 3.4 Evaluation of potential salinity‐tolerance genes

We investigated further potential salinity‐tolerance conferred by “suspect” genes identified by GWAS, first by examining the interaction among genes in the 12 common genomic regions by querying 43 physical interaction databases at http://genemania.org/ (Warde‐Farley et al., 2010) using *Arabidopsis* orthologues of the *Medicago* suspect proteins. We found that *Arabidopsis* AT1G04760, orthologous to Medtr2g028790 (Figure 6a VAMP726, synaptobrevin‐like protein), interacts with KAT3 (potassium channel in *Arabidopsis thaliana* 3; Figure S6a). Likewise, AT4G38510, an orthologue of Medtr7g081010 (Figure 6k, V‐type proton ATPase subunit B2), was found to have direct physical interaction with SOS2 (salt overly sensitive 2, CIPK24), which is known to be required for salinity tolerance in *Arabidopsis* (Figure S6b).

In an effort to further narrow down our list of suspect genes, we analyzed how these genes are regulated under salinity stress in *M. truncatula*, using published data from a study that employed hydroponics and relatively short‐term (up to 48 hr) salinity stress (Li, Su, Dong, & Wang, 2009). Additionally, in light of the fact that drought and desiccation, such as salinity, imposes osmotic stress on plant cells, we examined how our suspect genes responded to drought stress and during seed desiccation in *M. truncatula* (Verdier, Dessaint, Schneider, & Abirached‐Darmency, 2012; Zhang et al., 2014). Thirty nine of the 74 suspect genes were represented by microarray‐based (Affymetrix) data, and all but three of these genes were regulated under one or more of these three stress conditions (Table 2).

In addition to examining published gene expression data, we chose eight of the top suspect genes (Table 2 in bold) and performed qRT‐PCR analysis to determine if they respond at the transcript level to gradual salinity stress, as applied in our GWAS study. Gene selection for this experiment was based on SNP *P* values and rank, the position and density of enclosed SNPs, and gene function and expression patterns (Table 2, Table S3, and Figure 6). The eight genes selected included: one H^+^‐ATPase (Medtr7g081010); two putative transcription factors, a C2 domain protein (Medtr2g436900), and an mTERF protein (Medtr2g437020); two genes involved in vesicle trafficking, a non‐clathrin coat protein (Medtr2g028750), and a synaptobrevin‐like protein (Medtr2g028790); two genes involved in cellular redox homeostasis, an FAD‐linked oxidoreductase (Medtr3g009760), and a peroxidase family protein (Medtr4g046713); and a gene with unknown function (Medtr2g436940). Five *M. truncatula* lines were selected, three salt‐tolerant (HM091, HM010, and HM198 with salinity tolerance scores of 4.2, 3.8, and 3.5, respectively), and two salt‐sensitive lines (HM081 and HM152 with scores of 1.7 and 1.5, respectively).

**Figure 9:**
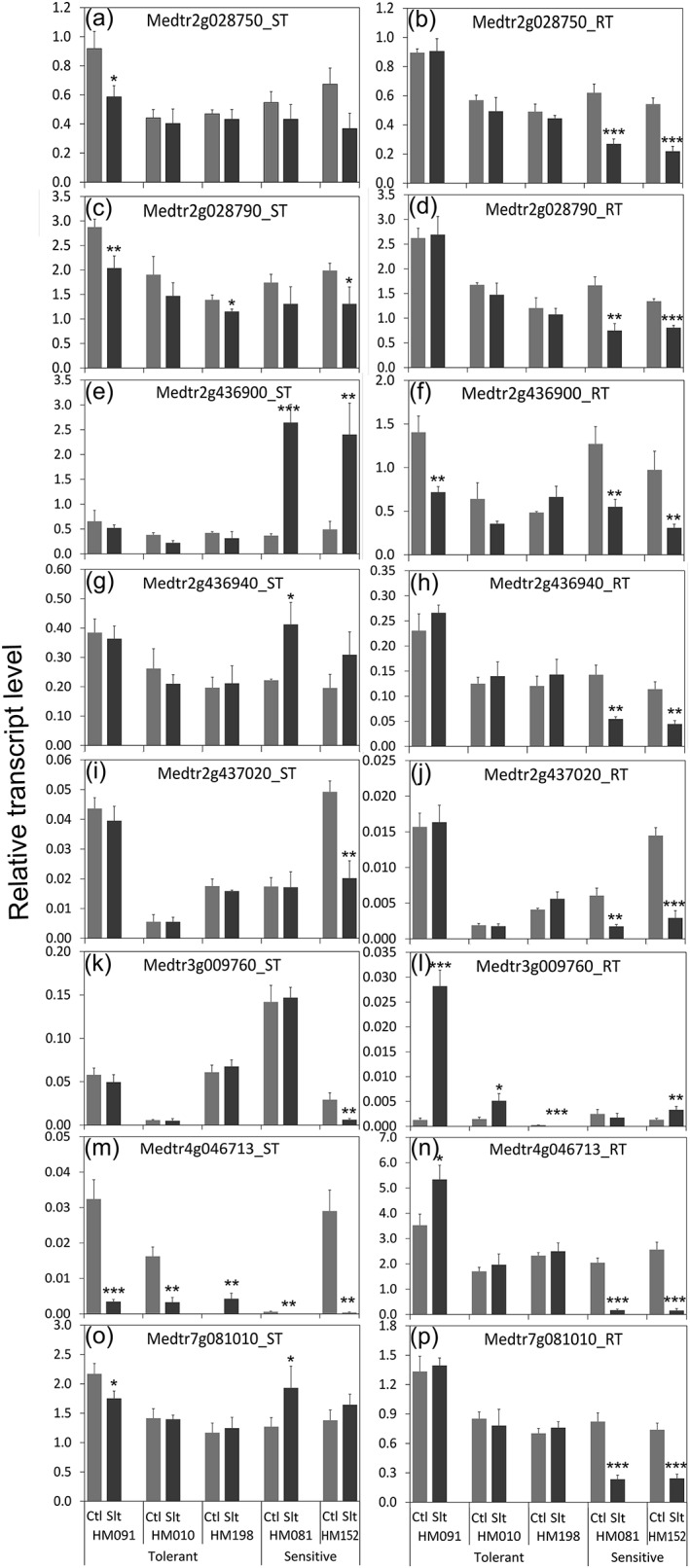
Transcript levels of potential causative genes in tolerant and sensitive genotypes. Relative transcript levels were determined by qRT‐PCR. The tolerance scores for HM091, HM010, HM198, HM081, and HM152 were 4.2, 3.8, 3.5, 1.7, and 1.5, respectively. Transcript levels are expressed relative to the mean of three housekeeping genes (*Ubiquitin‐Conjugating enzyme E2*, *Polypyrimidine Tract‐Binding protein* and *Ubiquitin*). *n* = 3. Error bars represent standard errors. Significance tests were between control and salinity stress treatments only in each genotype. Significat differences at *P < 0.05, **P < 0.01, and ***P < 0.001. Ctl: control. Salt: salinity‐treated

All eight of the selected genes were significantly regulated by gradual salinity stress in at least one of the lines, in the root, shoot, or both (Figure 9). The regulation patterns of the two genes involved in vesicle trafficking were similar; both were exclusively down‐regulated in the roots of the two sensitive lines (*P* < 0.01, Figure 9b,d). Similarly, salinity‐sensitivity was associated with transcript changes for Medtr2g436900 in the shoot (Figure 9e), Medtr2g436940 in the root and shoot (Figure 9g,h), Medtr2g437020 in the root (Figure 9j), Medtr4g046713 in the root (Figure 9n), and Medtr7g081010 in the root (Figure 9p). In contrast, the expression pattern of Medtr3g009760 (FAD‐linked oxidoreductase) was correlated to the salinity sensitivity in neither the shoot nor the root (Figure 9k,l).

In analyzing SNP variance in these eight genes, we extracted all the SNPs that reside within the genes and 1000 bp upstream and downstream of the start and stop codons, and selected those that varied consistently between tolerant and sensitive lines (Table S4). A total of 46 SNPs matching these criteria were identified, with Medtr7g081010 (H^+^‐ATPase) containing the most, whereas there were none in Medtr2g436940 (unknown protein). One missense mutation was found in Medtr4g046713 (peroxidase family protein), which caused an amino acid switch from Tyrosine to Serine. In addition, 10 SNPs occurred in introns and two in UTRs.

Finally, it is interesting to note that the profile of these 46 SNPs in the *M truncatula* A17 reference genome is highly similar to the two sensitive lines (HM081 and HM152), with only five being different (highlighted in Table S4). A17 was not used in the GWAS analyses but was characterized for salinity sensitivity as a check line; it has an average salinity sensitivity score of 2.1 and was, therefore, relatively sensitive to salinity stress (Figure S7).

## 4 DISCUSSION

### 4.1 Correlation among salinity responsive traits

Salinity is a complex abiotic stress, with ionic and osmotic stress components that trigger a variety of responses in plants. In the current study, we applied long‐term, gradual salinity stress to soil grown *M. truncatula* plants and characterized a wide‐range of physiological and biochemical traits in conjunction with GWAS. In the *M. truncatula* HapMap panel, we observed substantial variation in responses to salinity stress (Figure 1), and almost all of the traits characterized demonstrated a normal distribution (Figure 2). With multiple traits characterized, we were able to analyze the relationships among different traits as rarely done before. Sodium and chloride levels in leaves (*r* = −0.55, *P* < 0.001 for both) but not in roots (*P* > 0.2) were highly (negatively) correlated with salinity tolerance scores, with tolerant genotypes containing lower concentrations of sodium and chloride (Table 1). Therefore, it appears that maintaining low sodium and chloride concentrations in the shoot is an important strategy used by *M. truncatula* plants to survive and grow under salinity stress. This is consistent with earlier reports that photosynthetic organs are more sensitive than roots to high salt (Munns et al., 2006). The negative correlation between shoot sodium and chloride concentrations and salt tolerance has been reported previously in rice (Lin et al., 2004; Patishtan et al., 2017), Durum wheat (Munns et al., 2012), *Lotus* species (Sanchez et al., 2011), and barley (Nguyen et al., 2013). Interestingly, salt sensitivity does not seem to be linked to shoot sodium content in rapeseed (Wan et al., 2017; Yong et al., 2015).

Proline accumulation patterns under salinity stress were interesting. Earlier reports on the role of proline under plant salinity stress lack consensus, with some studies supporting its role as a protectant because it accumulates more in the tolerant than the sensitive genotypes, whereas other studies indicating that it may simply be a stress reporter when the opposite trend was found (Aziz et al., 1998; Heuer, 2010; Lacerda et al., 2003; Moftah & Michel, 1987). However, all previous studies used a small number of genotypes and only focused on the shoot. Here, we demonstrated that leaf proline levels are negatively correlated with tolerance scores (*r* = −0.59, *P* < 0.0001) and shoot water content (*r* = −0.65, *P* < 0.0001), whereas root proline levels are positively correlated with tolerance (*r* = 0.33, *P* = 0.0002; Figure 3; Table S2). In other words, salinity‐tolerant *M. truncatula* genotypes tended to accumulate more proline in the roots but less proline in the leaves than sensitive genotypes. This phenomenon has not been reported before and we postulate that it may reflect different roles of proline in roots and leaves. When plants are under salinity stress, the primary stress encountered by photosynthetically‐active leaves is redox stress (ROS) associated with insufficient ability to channel high‐energy electrons into anabolic pathways due to stomatal closure and reduced carbon fixation (Chaves, Flexas, & Pinheiro, 2009; Hossain & Dietz, 2016). Proline biosynthesis consumes electrons (NADPH), helping plants to cope with increased ROS production when stomata close (Hare & Cress, 1997). Salinity tolerant plants maintain vigor and photosynthesis for longer than sensitive plants under saline conditions, which likely keeps ROS levels relatively low in the tolerant plants (Ashraf, 2009). As a result, tolerant plants need not engage proline biosynthesis to the same extent as sensitive plants. Thus, the negative correlation between proline levels in leaves and overall salinity tolerance in *Medicago* indicates that proline in the leaf is not a primary tolerance mechanism, helping plants to avoid salinity stress, but rather a secondary line of defense to help plants survive when primary mechanisms fail.

The situation in roots appears to be quite different, where proline levels correlate positively with salinity tolerance. Rather than being subject to increased ROS associated with light‐energy harvesting without carbon fixation in leaves when stomata close, roots are prone to dehydration stress in saline soils. Proline biosynthesis in roots presumably contributes to overall osmolyte production in saline soils, which helps root cells take up the water necessary for transpiration, stomatal opening, photosynthesis, and so forth. By producing more proline in roots, salinity‐tolerant plants may be better able than sensitive plants to support shoot functions and carbon supply back to the root for further proline/osmolyte synthesis. In this sense, proline biosynthesis in the root may be a primary salinity tolerance mechanism in *Medicago*. Further studies are required to test this hypothesis, for instance by down‐regulating proline biosynthesis via RNA‐interference in transgenic “hairy roots” but not in shoots of *Medicago*. It would also be interesting to determine whether proline accumulates differentially in roots and/or shoots of plants with differential tolerance to other kinds of stress.

### 4.2 Identification and validation of suspect drought tolerance genes

To reduce false positives in our genome‐wide association studies, we used MLM and included population structure as co‐variants to counter bias due to any population structure. With this stringent MLM + Q + K analysis, we identified SNPs with *P* values as low as 10^−13^ (leaf K/Na ratio), which is far below the *P*‐value threshold after stringent Bonferroni correction (1.98 × 10^−8^ with 95% confidence). Taking advantage of the many salinity‐related traits that were measured, we focused on SNPs that were repeatedly linked to multiple traits, rather than simply selecting SNPs based on applying thresholds to individual traits. With this approach, 12 genomic regions with clear borders were identified (Figure 6, Table S3). A total of 74 genes and 214 SNPs were present in these 12 regions, with 94 SNPs within genes. Of these genes, 39 had corresponding transcript information in microarray data sets, and the majority were found to be either regulated by short‐term salinity stress or drought/desiccation stress in earlier studies (Li et al., 2009; Verdier et al., 2012; Zhang et al., 2014; Table 2). The tight LD among the top overlapping 214 SNPs (Figure 8) across multiple chromosomes indicates possible co‐evolution of the salinity tolerance genes. This phenomenon has been previously reported in human and mouse (Kulminski, 2011; Petkov et al., 2005). Note, however, that although multiple traits may map to the same genomic region, they are not necessarily associated with the same SNPs (Figure 6, Table S3). From GO enrichment analysis, we found that genes in the categories “response to stress” and “response to stimulus” are highly enriched with FDR < 0.001. Taken together, it is evident that the current GWAS studies identified a set of genes that are enriched in salinity/dehydration stress responses.

In examining the expression patterns of the eight top suspect genes by qRT‐PCR analysis of the five extremely tolerant and sensitive lines, we found significant regulation of these genes in response to gradual salinity stress (Figure 9). Association of salinity‐stress sensitivity with gene transcript level changes was evident in seven of these eight genes including one H^+^‐ATPase (Medtr7g081010), two transcription factors (Medtr2g436900, Medtr2g437020), two genes likely to be involved in vesicle trafficking (Medtr2g028750, Medtr2g028790), one gene involved in cellular redox homeostasis (Medtr4g046713), and one with unknown function (Medtr2g436940). The majority of these genes tended to have stable or increased expression under salinity stress in the tolerant lines but sharply decreased expression in the sensitive lines, especially in the root (Figure 9). The overall gene regulation patterns in the root bear similarity to those observed previously under short‐term salinity stress (Li et al., 2009) (https://mtgea.noble.org/v3/index.php). SNP variances correlating with salinity stress sensitivity were identified in these genes, including one that causes a missense mutation, and 12 causing modifications in the introns and the UTR regions (Table S4).

Vesicular trafficking has been demonstrated to play important roles in plant adaptation to salinity stress (Baral et al., 2015; Garcia de la Garma et al., 2015; Hamaji et al., 2009). Vesicle trafficking is likely to be involved in deployment of specific ion, water, or metabolite transporters to the cell or organellar membranes for ion or water homeostasis (Baral et al., 2015). Vesicle trafficking may also be involved in ROS signaling and facilitating plasma membrane area reduction during plasmolysis caused by osmotic stress (Baral et al., 2015; Leshem, Seri, & Levine, 2007). Here, we identified two genes that are involved in vesicle trafficking: one synaptobrevin‐like protein (Medtr2g028790), which is an R‐SNARE (Soluble N‐ethylmaleimide‐sensitive factor Attachment Protein) that mediates vesicle fusion, and one nonclathrin coat protein zeta1‐COP (Medtr2g028750), which is a component of the COPI coatomer that coats vesicles transporting proteins between the Golgi complex and the endoplasmic reticulum. Both genes had clear transcription reduction in the root under salinity stress in the sensitive genotypes but not in the tolerant genotypes in *M. truncatula* (Figure 9). Earlier studies demonstrated that modification of various SNAREs could either enhance or reduce salinity tolerance in *Arabidopsis* depending on the experimental set‐up (Hamaji et al., 2009; Leshem et al., 2007; Tarte et al., 2015). However, the involvement of non‐clathrin coat proteins in salinity stress responses has never been studied. Our GWAS results suggest a potential role of non‐clathrin coat protein zeta1‐COP in this process, which, thus, deserves further investigation.

Vacuolar H^+^‐ATPase (V‐ATPase) generates and maintains a proton gradient across the tonoplast that provides the driving force for secondary transport processes, including storage of excess ions in the vacuole under salinity stress (Silva & Gerós, 2009). Yeast two‐hybrid experiments showed that SOS2 (salt overly sensitive 2) interacts directly with V‐ATPase subunit B1 and B2 (AT4G38510, putative orthologue of Medtr7g081010), and mutant plants with reduced V‐ATPase activity were extremely salt sensitive (Batelli et al., 2007). In alfalfa, over‐expression of V‐ATPase subunit B from the halophyte, *Suaeda corniculata* (a putative orthologue of Medtr7g081010) resulted in plants more tolerant to salt and saline‐alkali stresses (Wang et al., 2016). Similarly, overexpression of the putative wheat orthologue (GenBank accession number: EF105343) of the *M. truncatula* V‐ATPase subunit B (Medtr7g081010) significantly improves germination rate under salinity and overall salt tolerance in *Arabidopsis* (Wang, He, Zhao, Shen, & Huang, 2011). In the current study, we found that the expression of V‐ATPase subunit B2 (Medtr7g081010) remained stable in roots of tolerant lines, but decreased significantly in sensitive lines. In addition, we identified multiple SNPs in the UTR region, the intron, and 1 kb vicinity of this gene that differentiate the tolerant from the sensitive genotypes. Work is ongoing to locate the causative SNP(s) underlying the differential regulation of this gene under salinity stress.

The importance of ROS scavenging and the two transcription factors (mTERF protein and C2 domain protein) in plant salinity stress responses have been reported in several previous studies (Abogadallah, 2010; Quesada, 2016; Robles, Micol, & Quesada, 2015; Xu, Leister, & Kleine, 2017; Yokotani et al., 2009). In the current study, we identified a peroxidase (Medtr4g046713) as a gene associated with salinity tolerance as it is the only predicted gene in a genomic region that harbors 26 SNPs linked to six distinct traits (Figure 6i). The *Arabidopsis* orthologue, rare cold inducible gene 3 (RCI3, AT1G05260), has been shown to increase salinity tolerance when overexpressed and to decrease salinity tolerance when mutated (Llorente, López‐Cobollo, Catalá, Martínez‐Zapater, & Salinas, 2002).

Differential regulation of suspect genes under salinity stress in tolerant versus sensitive lines implicates them in salinity responses, but does not establish their role in salinity tolerance. Further studies using hairy root overexpression and/or RNA‐interference, virus‐induced silencing (VIGS), stable transgenic, and *Tnt1* mutant plants are underway to investigate further the roles of these genes in plant salinity tolerance in *M. truncatula* and other species.

Apart from the genes discussed above that are linked to multiple salinity‐related traits, we are interested in genes associated with single traits that have homologs in other species that have been reported to play important roles in salinity tolerance (Table S3), including potassium channels/transporters, AAA family ATPases, cation/H^+^ exchanger, cation‐chloride cotransporter, LEA proteins, and a delta‐1‐pyrroline‐5‐carboxylate synthetase (P5CS), that is rate‐limiting for proline biosynthesis. As one example, the critical role of maintaining potassium homeostasis in plants during salinity stress has been intensively studied (Shabala & Pottosin, 2010). It has been shown that overexpression of a K^+^/H^+^ antiporter in tomato enhanced its salinity tolerance (Huertas et al., 2013).

Recently, GWAS of salinity stress‐related traits with relatively high precision have been performed in *Arabidopsis*, rice, and rapeseed (Al‐Tamimi et al., 2016; Kobayashi et al., 2016; Shi et al., 2017; Wan et al., 2017). We compared our GWAS results with these studies to identify potentially‐conserved salinity tolerance genes (orthologues). Among the top genes identified in various GWAS of salinity traits, only one, AT2G44480 (Table S5 in Kobayashi et al., 2016), which encodes a beta glucosidase, is homologous to a top gene identified in the current GWAS. Intriguingly, *M. truncatula* has three tandem repeats of this gene (Medtr4g066280, Medtr4g066330, and Medtr4g066340, Figure 6J), suggesting that gene expression levels may impact salt tolerance.

In summary, genome wide association studies (GWAS) of multiple physiological and biochemical traits related to salinity‐stress adaptation in *M. truncatula* were carried out using 2.5 million SNPs. Correlation analysis among the traits showed that salinity tolerance in the population is negatively correlated to sodium, chloride, and proline concentration in the leaf, while root proline levels were positively associated with salinity‐stress tolerance. GWAS analyses were performed with a mixed linear model, and 12 genomic regions that are associated with multiple traits each were identified; these regions harbor a total of 214 SNPs and 74 genes. These genomic regions were confirmed by a GWAS analysis using PC1 as a virtual trait, generated from principle component analysis of the 12 traits that are tightly correlated. Significant linkage disequilibrium was observed among these SNPs, possibly indicating co‐evolution of salinity tolerance alleles. Examination of gene expression of eight top suspect genes revealed associations between salinity tolerance and transcript level changes under salinity stress. Earlier functional studies on orthologues of two of the top eight genes (a vacuolar H^+^−ATPase and a peroxidase) confirmed their involvement in plant salinity tolerance in other species. Functional annotation of potential salinity tolerance genes points to the importance of transcriptional regulation, vesicle trafficking, and ROS scavenging under saline conditions.

## Supporting information


**Table S1.** Information on the 132 accessions that were used in the GWAS.
**Supplemental Table S2.** Correlation coefficients and association p values of all characterized salinity‐stress related traits.
**Supplemental Table S3.** List of 200 SNPs of the lowest p values for each trait.
**Supplemental Table S4.** SNPs reside inside as well as 1000 bp upstream or downstream of the eight genes that were examined by qRT‐PCR.
**Supplemental Table S5.** List of genes (SNPs) that had been identified in earlier GWAS of salinity‐stress related traits in rice (Al‐Tamimi *et al.,* 2016), Arabidopsis (Kobayashi *et al.,* 2016), and rapeseed (Wan *et al.,* 2017).
**Supplemental Table S6.** The list of primers that were used in the qRT‐PCR experiment.
**Supplemental Figure S1**. Example ion chromatography of line HM066 showing the difference in sodium and chloride in the leaf (a) and the root (b).
**Supplemental Figure S2.** Cladogram tree (neighbor‐joining) of the 132 lines that were used in the GWAS.
**Supplemental Figure S3.** Values of the Ln P(d) and Delta K as a function of the number of clusters (K).
**Supplemental Figure S4.** Quantile‐quantile (Q‐Q) plots for all the traits obtained by Mixed Linear Model (MLM) with (a) or without (b) Q value as a co‐variant, and General Linear Model (GLM) (c).
**Supplemental Figure S5.** GO enrichment of genes (1422 Arabidopsis orthologues) in the vicinity of the top 200 SNPs identified through GWAS of 17 traits (Table S3).
**Supplemental Figure S6.** Physical interaction networks of two selected genes in the top 12 genomic regions.
**Supplemental Figure S7.** Salinity stressed *M. truncatula* A17 plants.
